# The development of a network for community-based obesity prevention: the CO-OPS Collaboration

**DOI:** 10.1186/1471-2458-11-132

**Published:** 2011-02-24

**Authors:** Steven Allender, Melanie Nichols, Chad Foulkes, Rebecca Reynolds, Elizabeth Waters, Lesley King, Tim Gill, Rebecca Armstrong, Boyd Swinburn

**Affiliations:** 1 World Health Organization Collaborating Centre for Obesity Prevention, Deakin University, Burwood, Victoria, Australia; 2 British Heart Foundation Research Group, Department of Public Health, University of Oxford, Oxford, UK; 3 The McCaughey Centre, Melbourne School of Population Health, University of Melbourne, Melbourne, Australia; 4 Prevention Research Collaboration, University of Sydney, Sydney, New South Wales, Australia

## Abstract

**Background:**

Community-based interventions are a promising approach and an important component of a comprehensive response to obesity. In this paper we describe the Collaboration of COmmunity-based Obesity Prevention Sites (CO-OPS Collaboration) in Australia as an example of a collaborative network to enhance the quality and quantity of obesity prevention action at the community level. The core aims of the CO-OPS Collaboration are to: identify and analyse the lessons learned from a range of community-based initiatives aimed at tackling obesity, and; to identify the elements that make community-based obesity prevention initiatives successful and share the knowledge gained with other communities.

**Methods:**

Key activities of the collaboration to date have included the development of a set of Best Practice Principles and knowledge translation and exchange activities to promote the application (or use) of evidence, evaluation and analysis in practice.

**Results:**

The establishment of the CO-OPS Collaboration is a significant step toward strengthening action in this area, by bringing together research, practice and policy expertise to promote best practice, high quality evaluation and knowledge translation and exchange. Future development of the network should include facilitation of further evidence generation and translation drawing from process, impact and outcome evaluation of existing community-based interventions.

**Conclusions:**

The lessons presented in this paper may help other networks like CO-OPS as they emerge around the globe. It is important that networks integrate with each other and share the experience of creating these networks.

## Introduction

Overweight and obesity is one of the major threats to the health of Australians, as it affects a significant proportion of the population (about 60% of adults and 25% of children) and is a key risk factor in the development of chronic diseases such as type II diabetes, coronary heart disease and many cancers [1]]. Obesity prevalence has risen rapidly in recent decades and also disproportionately affects people from socially and economically disadvantaged backgrounds [2-7].

The behavioural patterns contributing to high rates of obesity include increased consumption of high energy density foods and beverages, low consumption of fruit and vegetables and a shift to less active transport and more sedentary leisure time activities [8-10]. Obesity develops in a social and environmental context in which facilities, policies, economic factors and socio-cultural influences promote these behaviours [11,12]. Thus, it is likely that education and treatment approaches alone will not be sufficient to reverse the obesity epidemic or its socio-economic gradient [13].

A number of countries have initiated Community-Based Obesity Prevention Initiatives (CBOPIs) as part of their attempts to address the obesity epidemic. The rationale for such an approach is multi-faceted and strong [14]. The strengths of CBOPIs to prevent obesity include the ability to influence a wide range of determinants of nutrition and physical activity behaviours and the ability to utilise and strengthen existing community assets and capacity in multiple community-settings [15,16]. Whole-of-community interventions are consistent with a socio-ecological understanding of obesity, involving multiple spheres of influence, and are a crucial complement to individual-focused activities. In addition, interventions which focus on promoting healthier environments are likely to be more equitable than those primarily using educational approaches [17,18]. However, CBOPIs themselves can only be part of a more comprehensive response to obesity which would include national (and even international) policies and actions - especially in creating healthier food supply and marketing systems. Internationally, Ensemble prevenons l'obesitie des enfants (EPODE) is one of the first CBOPIs to show an effect on childhood obesity. In a pilot study, this whole of community intervention showed a significant decrease in obesity for intervention compared to comparison towns after 12 years [19] and has now been extended across France, Spain and Belgium [20].

In a number of other countries, including the USA, Australia and New Zealand, evaluation of demonstration projects in community-based obesity prevention have recently begun to show promising results, particularly in children [16,17,21,22]. Although results to date have been relatively modest in most cases, small changes affecting large populations will result in significant population health gains [23].

There are as many as 12 large-scale (highly evaluated) CBOPI demonstration projects around Australia, many of which are ongoing. Many other similar large scale programs are planned or underway. There are also countless smaller, setting-specific interventions (e.g. schools, pre-schools, worksites). This range of projects is developing the evidence and expertise about what works and what does not work in promoting healthy eating and physical activity and sharing this information is important for national and international audiences. A central network could ensure that community projects are informed by the highest quality, best available knowledge, evidence, expertise and experience collated from existing CBOPI. A national collaboration may help to reduce the unnecessary costs of duplication of effort in activities such as extracting evidence from the literature, designing programs, developing measurement tools, creating intervention resources.

In this paper we introduce the Australian COmmunity-based Obesity Prevention Sites (CO-OPS)Collaboration, with the view to informing the establishment of similar collaborative networks worldwide. We introduce CBOPIs and describe the development and form of the CO-OPS network. The domains of activity for the CO-OPS Collaboration are described in detail and include; establishing the depth and breadth of CBOPI in Australia; establishing a collaborative network of these CBOPI; developing a set of best practice principles for CBOPI; promoting evidence, evaluation and analysis in practice; establishing a practice relevant evidence base; and knowledge translation and exchange (including dissemination).

### Collaboration of Community-based Obesity Prevention Sites - The CO-OPS Collaboration

In 2007, three Australian universities, led by Deakin University and in collaboration with the University of Melbourne and the University of Sydney, received four years of funding from the Australian Federal Government Department of Health and Ageing to create the CO-OPS Collaboration. The core aims of the Collaboration are to:

• identify and analyse the lessons learned from a range of community-based initiatives aimed at tackling obesity, and

• identify the elements that make community-based obesity prevention initiatives successful and share the knowledge gained with other communities

The structure of the CO-OPS Collaboration incorporates a steering committee, advisory committee, secretariat and the broader membership of practitioners, researchers and others with an interest in community-based obesity prevention. The secretariat is the central (staffed) structure of the Collaboration, with expertise in public health research, program evaluation, community engagement and knowledge translation and exchange.

### Defining the boundaries - what is community-based obesity prevention?

Initiatives were considered to be 'obesity prevention' if they intended to promote healthy weight or prevent unhealthy weight gain and/or promote healthy eating or physical activity in a manner that could be expected to influence energy balance in communities or populations. Defining whether an initiative is 'community-based' is more problematic. Definitions of 'community' include common themes of geography, social interaction (mutual support, a sense of belonging, interlinked networks) and/or common ties (beliefs, activities, culture, interest, experience, political and social movements, etc). 'Community-based' may be as broad as any program that involves community engagement or participation [24]. For the CO-OPS Collaboration, 'community-based' means projects that focused on whole communities (however defined) or which intervene at a population level. This excludes programs specifically or exclusively focused on individual education or behaviour change. Community-based programs are most often delivered at or through local community settings (e.g., schools, workplaces, community centres, etc), although they may be centrally organised but locally delivered. Clinical activities in the community are excluded as they are individual level activities delivered in the community rather than population-focused promotion of health [25].

### How many initiatives are there in Australia?

A 'top-down' snowball sample was employed to develop the CO-OPS network and better understand current community-based obesity prevention in Australia. Top-down recruitment involved identification of CBOPIs by state government health departments and snowball sampling asked participants in the survey to identify other relevant initiatives known to them.

Of the 78 initiatives identified, around a quarter of projects were focused on the entire spectrum of ages (24%), while among those that targeted specific groups, the largest proportion of projects targeted primary school children (37%) and adults (33%). The projects ranged from less than 100 participants (11% of projects) to interventions in communities of more than 50,000 (26%).

### Establishing a collaborative network

The CO-OPS Collaboration was established to provide members access to networks of health professionals, researchers and government employees all interested in community-based obesity prevention. CO-OPS as a 'community of practice' grew from networks of known individual practitioners working within existing initiatives to a community of more than 1300 professional members representing practitioners and researchers in CBOPI across Australia and internationally. CO-OPS is the 'community of practice' for those working in community-based obesity prevention in Australia. CO-OPS extends beyond an information sharing network by intentionally creating new knowledge in collaboration. As a community of practice this new network has created a system with qualities and capacities beyond those present within individuals acting separately [26].

CO-OPS can be conceived of as a 'community of practice' providing a forum for people to "share a concern, a set of problems, or a passion about a topic, and who deepen their understanding and knowledge of this area by interacting on an ongoing basis"[27] Communities of practice comprise three essential elements. Firstly they have a *domain*: this is the concern, issue or passion, and for CO-OPS the domain is obesity prevention (including promotion of nutrition and physical activity); secondly they have a *community*, these are the people who care about the domain (obesity prevention), they share and combine ideas, thoughts and questions, for CO-OPS the community consists of those who have become members of the website, those who attend training or workshop events and those working with CBOPI who are yet to join CO-OPS; thirdly they have practices, practices help the community (CO-OPS members) to develop new knowledge about the domain (obesity prevention). For CO-OPS, practices include the Best Practice Principles, Evidence Summaries, National Workshop presentations and resources from member (community) projects. Practices can be written or codified and they can also be beliefs or stances i.e. obesity requires interventions at the community level.[27]

Communities of practice have varying levels of participation by members from a core group (CO-OPS Secretariat) through active (Advisory Committee members, those regularly contributing to newsletter) and occasional participants (members who assist to organise training, provide resources for the website, contribute to case studies, attend the National Workshop, submit requests for assistance through the website) to peripheral (network members who have chosen to sign up to receive the newsletter however do not contact or contribute to CO-OPS and those who know of CO-OPS however have decided not to join) and transactional participants (these are people, organisations or contractors who provide services). Levels of participation are not static and CO-OPS members easily shift between roles depending on the need of the network and the expertise available amongst its members [28].

**Figure 1:**
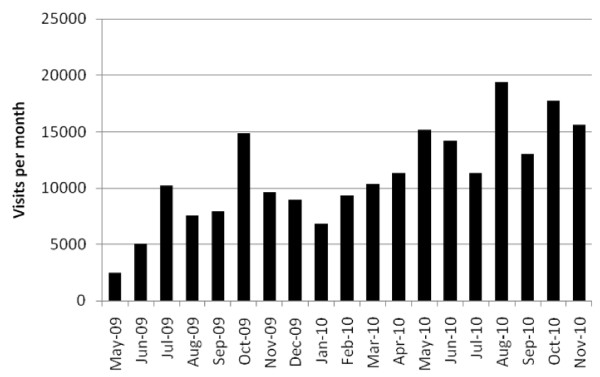
**Web site page visits to the CO-OPS Collaboration web site, May 2009 to November 2010**.

Success in the establishment of the collaborative network to date has been demonstrated by the rapid growth in membership from an initial 30 founding members to now include over 1300 professionals (end 2010) who are active in research or practice relating to community-based obesity prevention. Of equal importance is the breadth of membership: members of the network include representatives from federal government departments, all state and territory governments (various departments, including health, human services, transport and planning) 52 different local government authorities, 42 community health organisations, 23 non-governmental organisations (NGOs) and 22 Australian universities. The size of the network and the interactions that network members have with the Collaboration is approximated by the number of visits to the CO-OPS website. Total numbers of visitors to the website, by month, are shown in figure 1.

### Establishing Best Practice Principles for Community-based Obesity Prevention

A set of "Best Practice Principles for Community-based Obesity Prevention Initiatives" (BPP) was developed as a core document to guide the activities of the CO-OPS Collaboration and provide an important reference and guide for its members[24]. The BPP were developed by combining information obtained from a two-stage literature review with extensive consultation to elicit the experience and knowledge of stakeholders in well-developed and well-evaluated obesity prevention programs across Australia. Following extensive consultations and reviews, 22 Best Practice Principles were developed in five key areas. Details of the development, content and application of the principles are published elsewhere[24], however the principles are summarised in table 1.

**Table 1:** Best Practice Principles for Community-based Obesity Prevention

**Community engagement**	**Evaluation**
C1. Approach to community engagement	E1. Evaluation framework and approach
C2. Community analysis	E2. Evaluation plan
C3. Implementation partnerships	E3. Data collection and management
C4. Program sustainability and community capacity	E4. Evaluation context
	E5. Active dissemination
**Program design and planning**	**Implementation and sustainability**
P1. Problem analysis and program focus	I1. Consumer testing of messages, resources and approaches
P2. Framing of the problem	I2. Quality implementation and monitoring
P3. Planning context	I3. Adaptations and responsiveness
P4. Evidence and innovation	
P5. Theory of change/change process	
P6. Feasibility	**Governance and accountability**
P7. Program plan	G1. Explicit funding sources
P8. Target groups	G2. Program management structure

As a practical resource for members of the CO-OPS network, the BPP were published as three connected resources. The first BPP resource, the *Outline and Rationale of Best Practice Principles for Community-based Obesity Prevention *is the most detailed section. It presents principles, supporting literature and commentary on specific issues related to the application of the principle to community-based obesity prevention. The second resource, *Guiding Questions for Community-based Obesity Prevention*, provides a set of practical guiding questions relating to each of the principles. These questions are designed to assist in applying the principles and are intended as a learning tool for practitioners. The third resource, *Short Guiding Questions for Community-based Obesity Prevention*, presents a simple checklist and easy introduction to the full documentation of BPP and the guiding questions. The documents are available for download from http://www.co-ops.net.au. To date, the response to the BPP has been very positive, and the resource has been downloaded over 800 times, in addition to over 1500 hard copies distributed through professional development sessions and networking opportunities. This clearly demonstrates the reach of the BPP and a strong demand for such a resource. Elements of the BPP have been incorporated into the quality framework for a very large federally funded community-based obesity prevention initiative (Healthy Communities) and preliminary qualitative feedback indicates that the BPP have been employed in a range of settings to guide planning, implementation and evaluation of programs. A more comprehensive review of the use and implementation of the BPP is planned for early 2011.

### Promoting evidence, evaluation and analysis in practice

A core aim of the CO-OPS Collaboration is to promote knowledge generation and translation among community-based obesity prevention initiatives and thereby reduce duplication of effort. Included in this aim is the need to promote high quality evaluation of existing programs and dissemination of these results. Key activities to promote evidence quality and analysis include identifying robust tools and methods for measurement and exploring the potential of data management processes for meta-analyses. Creating evaluation and data management support service for projects will be very important in promoting evaluation results which can be translated to other settings and inform future practice and to enhance capacity within community initiatives.

Currently, very little is known about the scope and rigour of evaluations being conducted in the majority of CBOPIs and initial consultation with community based professionals including members of the CO-OPS Collaboration identified the greatest need for skill development was in evaluation. The limited academic literature indicates little agreement about evaluation design, methodology (particularly data collection) and as a result limited consistency or comparability across evaluations. High quality evaluation and clear evidence of effectiveness is needed for community-based interventions to progress. CO-OPS is beginning to address this need through:

• identification and promotion of high quality evaluation tools and instruments for consistent and comparable data collection,

• provision of support and advice to practitioners evaluating CBOPI,

• dissemination of evaluation findings to the network, to promote successes and reduce repetition of failures, and

• meta-analyses of CBOPI evaluation findings.

### Establishing a practice relevant evidence base

A consistent finding of national consultations was the need for accessible and practice relevant summaries of knowledge and evidence summaries. In response, CO-OPS supported the development of three practitioner-led evidence summaries. The process involved workshops to develop an 'answerable' research question with practitioners, discussion of the best ways in which to present evidence summaries, a review of systematic reviews related to the question as defined (guided by the results of the workshops) and publication and dissemination of the evidence summary both as hard copies and via the project web site. The topics of the three evidence summaries developed to date are:

• Remote and rural issues in the prevention of obesity for pre-adolescents and adolescents

• Achieving equity in community-based obesity prevention interventions for children and adolescents

• Considerations regarding harm minimisation for obesity prevention policies and programs for pre-adolescents and adolescents

Local government is an important platform for the implementation of CBOPIs and this is an increasing level of activity in this area. The role of local government in obesity prevention was emphasised by the recent commitment of Australian governments to the Healthy Communities Initiative, which charged local government with delivering nutrition and physical activity promotion through community-based programs. A second round of evidence summaries were developed to clearly understand the role of local government in obesity prevention and to provide evidence relevant to this setting. Consultation with sites in the CO-OPS network was conducted to understand the capacity of local government in using research evidence by developing and evaluating an 'evidence tool kit'. The purpose of the toolkit was to strengthen capacity of local governments to use research evidence when formulating obesity prevention policies and programs at the local level.

Australia does not have a national, uniform system for monitoring overweight and obesity among children. Such a system would allow examination of trends in obesity over time and also provide information for evaluation of program effectiveness. CO-OPS has commissioned a series of reports to inform the development of a monitoring system, addressing; *monitoring system design; ethics assessment; and, feedback systems of monitoring information*.

In addition to the six newly developed evidence summaries and the three reports described above, the CO-OPS resource library currently includes a wide variety of resources relevant to evidence-based practice, including systematic reviews, validated evaluation tools and project reports from large and well-evaluated programs (84 resources at the end of 2010). The evidence summaries have been among the most popular resources downloaded form the CO-OPS website, surpassed only by the BPP. The growth in usage of the CO-OPS website (figure 1) demonstrates that it is becoming established as a key resource for practitioners.

### Knowledge translation and dissemination

A key function of the CO-OPS Collaboration is to facilitate knowledge translation and exchange among members of the network. Specific activities include identifying and addressing knowledge gaps, translation of research evidence into practice-relevant knowledge and evidence-based recommendations, sharing of lessons learned in practice between initiatives, and collating and disseminating both research- and practice-based evidence.

Available evidence suggests that the most effective knowledge translation occurs when recipients feel they are competent and confident in the use of evidence and *a priori *learnings, accompanied with easily accessible information, key messages, a facilitated network and access to a knowledge broker role. This is the approach that the CO-OPS Collaboration takes by having a knowledge broker as a core part of the Secretariat. Additional strategies used by CO-OPS to encourage successful translation of knowledge include:

▪ working with practitioners working in CBOPIs to identify knowledge needs,

▪ sourcing and developing resources, evidence summaries and other knowledge resources for the sites, and

▪ 'translating' the knowledge for CO-OPS sites and a wider audience through a variety of approaches including websites, emails, newsletters, training programs and site visits,

As a national collaboration in a geographically very large country, internet-based resources and services are a key component of CO-OPS' communication with the network. The CO-OPS website http://www.co-ops.net.au includes electronic access to all new resources developed for CO-OPS (including those described above), a number of discussion forums, a searchable database of existing CBOPI in Australia and a searchable resource library providing access to a wealth of relevant information and resources for CBOPI, including evaluation tools and protocols, project reports and systematic reviews of the evidence.

The website is the practical repository for knowledge received and created by CO-OPS members and project staff. Contributions to these resources and knowledge come from members in the form of evaluation tools, program designs, academic journals, training presentations and external sources. As a network it is imperative that CO-OPS codify and disseminate knowledge of relevance to its members to ensure it retains its position of importance in members work and professional estimations. CO-OPS achieves this by using data such as web-statistics to monitor what internet resources are accessed; to assess which parts of its electronic newsletter are most visited; through discussion with members on their training needs; through its governance structure of an Advisory and Steering Committee; and tracking requests for assistance. Evidence from these data sources indicates that resources are well used and highly accessed, particularly those that relate to case studies of best practice (typically >120 downloads per individual case study and over 400 downloads and 700 hard copies for the collection of case studies collated from the 2009 National Workshop) and evidence syntheses (>570 downloads in addition to >600 hard copies distributed).

Since its establishment, members of the CO-OPS secretariat have conducted 26 professional development sessions across Australia, for more than 650 practitioners, in addition to over 70 other presentations to a variety of national and international audiences. There has been high demand for these activities, with sessions regularly being over-subscribed well in advance.

In addition, it appears from the available data that rates of access and use of the online (website) resources are heavily dependent upon active knowledge translation activities, including the dissemination of newsletters highlighting available evidence and resources, but particularly the networking and professional development events run by CO-OPS. These events, which often introduce available resources and guide members in how to access and use them, appear to facilitate members to engage in a more in-depth manner with resources and evidence. The levels of website usage shown in figure 1 reflect key events, particularly the national workshops held in October 2009 and 2010 and a series of state-based workshops around February to March and May to June 2010 (the spike in August 2010 resulted from a variety of online promotion activities and presence of links to CO-OPS in the communications of other professional networks).

As CO-OPS grows in member numbers, available resources and stature it is becoming and needs to continue to become increasingly sophisticated in how it reaches and works with members to steward and disseminate knowledge. CO-OPS is enhancing the meaning and quality of its interactions with members through increasing its modes of assistance, depth of discussion and member case studies. This is achieved by working with members and non-members to identify the specific training required to better tailor the information, case studies and type of training it delivers. CO-OPS is also moving to expand the availability of its training and the discussions within these sessions to a broader audience and to continue the discussions beyond traditional face-to-face training towards electronic options including web forums. These expanding, more inclusive and increasingly tailored opportunities to steward and disseminate knowledge will see CO-OPS develop as a network and progress through the various stages of development of a community of practice.

## Discussion

The establishment and development of the CO-OPS Collaboration provides an important model for support and knowledge exchange for community-based obesity prevention. Emerging evidence from successful community-based efforts to prevent obesity suggests that a significant level of centralised support is necessary, especially in relation to evaluation and evidence. The widely adopted EPODE model of intervention, which originated in France, began with a funding structure allocating 50% of funds to interventions and 50% to central support and evaluation. Currently EPODE costs around €2 per capita from the local government and 20c is used centrally (10%) for evaluation (personal communication). Australian demonstration programs in community-based obesity prevention have also shown the importance of strong support and central infrastructure. The CO-OPS Collaboration further expands this notion to bring together obesity prevention initiatives intervening at the community level from across the country, facilitating knowledge exchange and translation between initiatives of varying size, scope and focus. This ensures that lessons learned are shared and translated to other relevant contexts as well as providing support to practitioners who may be intervening in novel ways, with less common target groups or who may be geographically isolated.

A number of research-based networks have been established internationally to bring together expertise on obesity prevention, especially for children. There are fewer examples for models of collaboration to build a network of professionals in practice, policy and research. Notable recent exceptions include the Canadian Obesity Network http://www.obesitynetwork.ca/ and the Canadian Partnership Against Cancer Coalitions Linking Action and Science for Prevention http://www.partnershipagainstcancer.ca/priorities/primary-prevention/strategic-initiatives/coalitions. The expertise in implementing community-based initiatives which is held in the non-academic sector is vast and valuable. A strength of the CO-OPS Collaboration is this breadth of stakeholder representation, the model of learning and exchange between academic and non academic sectors, and the recognition of a variety of experiences and types of evidence and information.

Our experience in establishing the network and consulting widely with a range of stakeholders has provided a number of important lessons about the needs of practitioners working in CBOPI. Chief among these is the need for professional networks and for support in planning and conducting program evaluation and using and contributing to current research.

Key success factors in the process of setting up the collaboration have been the political, multi-level government policy, and commonwealth funding support and the overarching governance arrangements, each of which have helped ensure a systems integrated approach. Operationally, this resulted in the establishment of a secretariat, a steering committee, an advisory committee and defining the form and function of the network through consultation with stakeholders. The input of key stakeholders from government, research and practice in the steering and advisory committees has been crucial to guiding the direction of CO-OPS secretariat activities and developing the network. Another key factor in successful developments to date has been a secretariat and partner university collaborators with the skills and capacity to provide advice and support to network members in knowledge translation, program evaluation and training and supporting networking and training.

Accommodating the diversity of CBOPI across in Australia, from very large, well funded programs with academic support through to small community-health led activities in single communities has proved a major challenge. The geographical size (and attendant remoteness of some areas) of Australia and the cultural diversity of communities provide a second challenge. The CO-OPS secretariat have endeavoured to overcome this in part by regularly travelling to each state and territory, including outside of capital cities, for consultation, networking activities and professional development, and in part by ensuring that as many of the resources and support services as possible are available online.

The CO-OPS Collaboration is now at the forefront of community-based obesity prevention in Australia, bringing together research, practice and policy. As the policy and practice environment continues to evolve, it will be critical to ensure that CO-OPS retains its relevance and adapts to new challenges, opportunities and initiatives. The greater the critical mass of action in community-level obesity prevention grows, the greater the need for a central support and knowledge translation network will become. Significant gaps remain in knowledge and evidence related to CBOPI. An important future function of CO-OPS will be to facilitate further evidence generation and translation, including combining findings from a range of initiatives to draw broader conclusions about process, impacts and outcomes of CBOPI. Given the global significance of the obesity epidemic, the natural next stage to share experiences and develop the evidence will be to build international networks along the CO-OPS model, bringing together stakeholders in research, practice and policy.

## Conclusion

Community-based interventions are a promising approach and an important component of a comprehensive response to obesity. The establishment of the CO-OPS Collaboration is a significant step toward strengthening action in this area, by bringing together research, practice and policy to promote best practice, high quality evaluation and knowledge translation and exchange.

The lessons learned from the CO-OPS Collaboration provide valuable insight into the development of national collaborative efforts and should be taken into consideration when establishing similar national partnerships and in combined international community based efforts to prevent obesity.

## Competing interests

The authors declare that they have no competing interests.

## Authors' contributions

SA, BS, MN, CF were involved in all activities reported within the paper. EW, RA led the evidence summary work presented in this paper. LK and TG led the work described under best practice principles. SA, MN, CF, RR led the writing of initial drafts of this paper. All authors have been involved in drafting the manuscript or revising it critically for important intellectual content. All authors have given final approval of the version to be published

## Pre-publication history

The pre-publication history for this paper can be accessed here:

http://www.biomedcentral.com/1471-2458/11/132/prepub
